# Oropharyngeal Carcinoma Presenting as a Large Cervical Mass With Impending Airway Obstruction

**DOI:** 10.7759/cureus.88206

**Published:** 2025-07-17

**Authors:** Yasutaka Yanagita, Kiyoshi Shikino

**Affiliations:** 1 General Medicine, Chiba University Hospital, Chiba, JPN; 2 Community-Oriented Medical Education, Chiba University Graduate School of Medicine, Chiba, JPN

**Keywords:** acute airway obstruction, cervical lymphadenopathy, emergency tracheostomy, oropharyngeal cancer, oropharyngeal carcinoma, squamous cell carcinoma (scc), tobacco-related malignancy

## Abstract

A middle-aged woman presented with progressive pharyngeal symptoms and an enlarging neck mass, eventually diagnosed as human papillomavirus (HPV)-negative oropharyngeal squamous cell carcinoma (OPSCC). Due to impending airway obstruction, an emergency tracheostomy was performed. Histopathological examination confirmed squamous cell carcinoma, leading to a final diagnosis of non-HPV-associated OPSCC, a rarer and more aggressive subtype. Despite receiving palliative radiotherapy for symptom control, the disease progressed aggressively, and the patient was ultimately transitioned to best supportive care.

OPSCC arises from the mucosal epithelium of the oropharynx. Early symptoms such as persistent throat discomfort, dysphagia, and cervical lymphadenopathy are relatively characteristic of oropharyngeal tumors. Tumor progression can cause critical airway obstruction depending on the lesion’s size and location. In this report, we present a case of OPSCC that required emergency airway management due to the risk of airway obstruction. This case underscores the importance of early diagnosis and airway management in patients with progressive pharyngeal symptoms and high-risk features.

## Introduction

Oropharyngeal squamous cell carcinoma (OPSCC) is a malignant tumor arising from the mucosal epithelium of the oropharynx, including the base of the tongue, tonsils, soft palate, and pharyngeal walls. Major risk factors include chronic tobacco use and alcohol consumption [1]. Early symptoms such as persistent throat discomfort, dysphagia, and cervical lymphadenopathy are relatively characteristic of oropharyngeal tumors. Tumor progression may cause critical airway obstruction depending on the lesion’s size and location. Upper airway obstruction during tumor progression occurs in approximately 3%-20 % of patients with head and neck malignancies, but it is especially uncommon in patients with OPSCC and predominantly seen in advanced stages or aggressive human papillomavirus (HPV)-negative subtypes [2]. OPSCC is clinically staged using the TNM classification, which evaluates the size of the primary tumor, regional lymph node involvement, and distant metastasis. The standard treatment for localized OPSCC often includes a combination of radiotherapy and chemotherapy, particularly for stage III-IV disease, depending on the patient’s performance status and extent of metastatic spread. Contrast-enhanced computed tomography (CECT) findings, such as irregular nodal borders and central necrosis, are important indicators of malignant lymph node involvement [3].

Clinically, OPSCC can be categorized into HPV-positive and HPV-negative OPSCC. HPV-positive tumors have become more prevalent in developed countries and generally respond better to treatment, with a five-year survival rate of over 80%. In contrast, non-HPV-associated OPSCC, which is more often linked to smoking and alcohol use, remains more aggressive and has a poorer prognosis, with survival rates below 50%. Globally, the incidence of OPSCC is estimated at 1.1 per 100,000 individuals annually [2]. The proportion of non-HPV-associated OPSCC cases is declining in the post‑vaccine era, but this disease persistently shows significantly worse prognosis and lower five‑year survival (<50%) compared to its HPV-associated counterpart (>80%) [2].

In this report, we present a case of OPSCC in a middle-aged woman who required emergency airway management due to the risk of emergent airway obstruction [4,5]. This case highlights the importance of early diagnosis, recognition of high-risk features, and urgent airway management.

## Case presentation

A 51-year-old woman experienced discomfort in her pharyngeal region for six months, accompanied by progressively worsening symptoms such as dysphagia, odynophagia, hoarseness, and dyspnea, particularly when bending forward or tilting her neck. She noticed swelling on the left side of her neck that had appeared insidiously and had progressively grown prominent over two months, alongside a significant weight loss of 10 kg within six months. She had a smoking history of 30 pack years (20 cigarettes/day for 30 years) and occasionally consumed alcohol. The patient had no significant medical history, no known exposure to tuberculosis, and was not taking any regular medications. She had no notable family history. She had not presented to any clinic for her throat discomfort until respiratory distress became evident.

**Figure 1:**
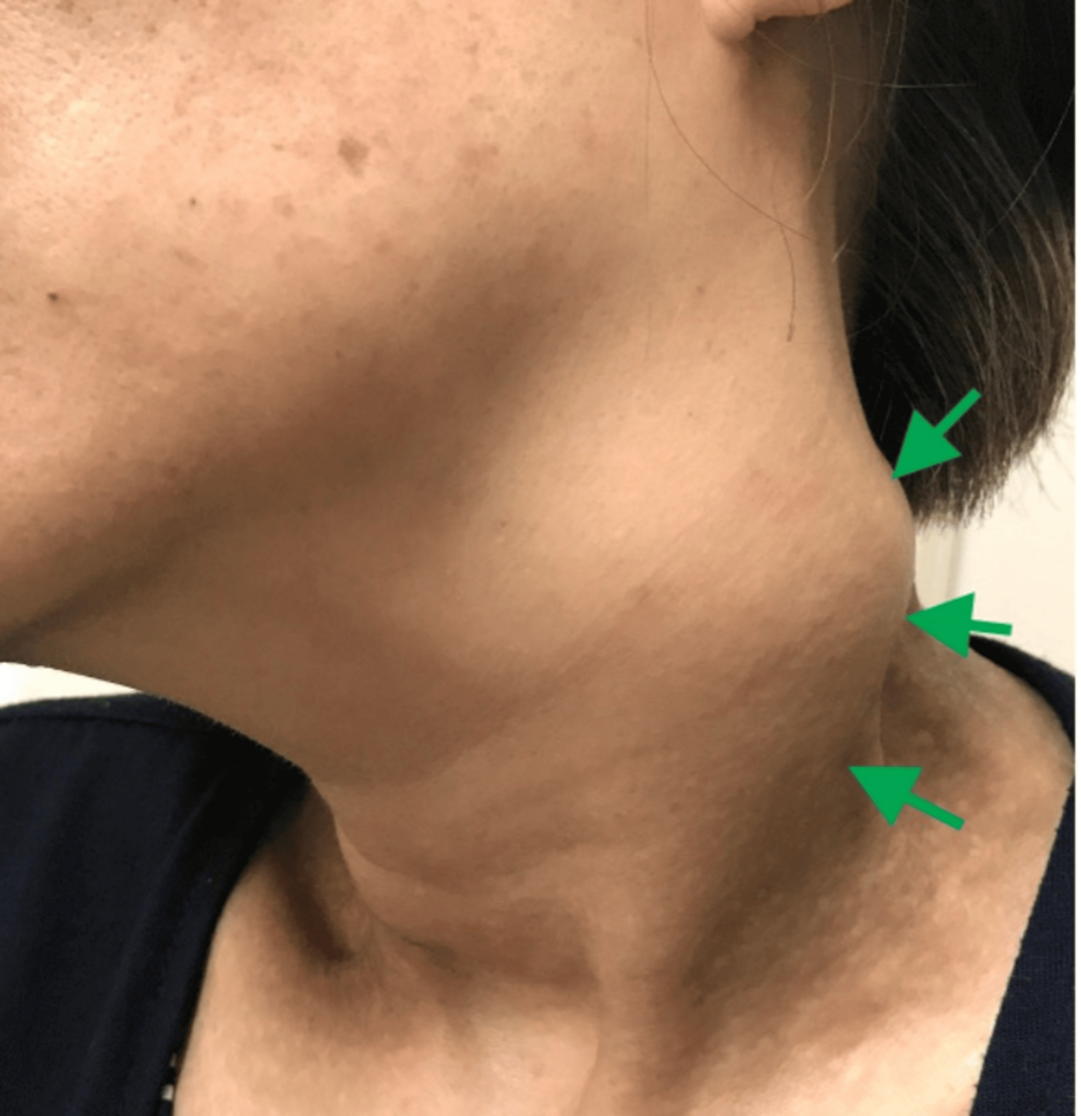
Massive lymphadenopathy

The physical examination showed multiple bilateral neck lymphadenopathies and a palpable, firm, non-mobile mass on the left side of the neck, measuring ~4 cm in diameter (Figure 1). Auscultation revealed stridor, labored breathing, and resting oxygen saturation within the normal range. With neck flexion, the stridor worsened, and oxygen saturation decreased.

Blood tests revealed mild normocytic anemia, elevated inflammatory markers, and signs of hepatic dysfunction, which were consistent with systemic inflammation and possible metastatic involvement. These findings supported a suspicion of advanced malignancy (Table 1).

**Table 1:** Laboratory results

Test	Result	Reference range
Hematology		
Hemoglobin	11.2 g/dL	Female: 11.5-15.0 g/dL
Mean corpuscular volume	102.5 fL	80-100 fL
Biochemistry		
Lactate dehydrogenase	134 IU/L	120-240 IU/L
C-reactive protein	8.3 mg/dL	<0.3 mg/L
Alkaline phosphatase	607 U/L	100-350 U/L
Iron	24 μg/dL	Female: 50-170 μg/dL

**Figure 2:**
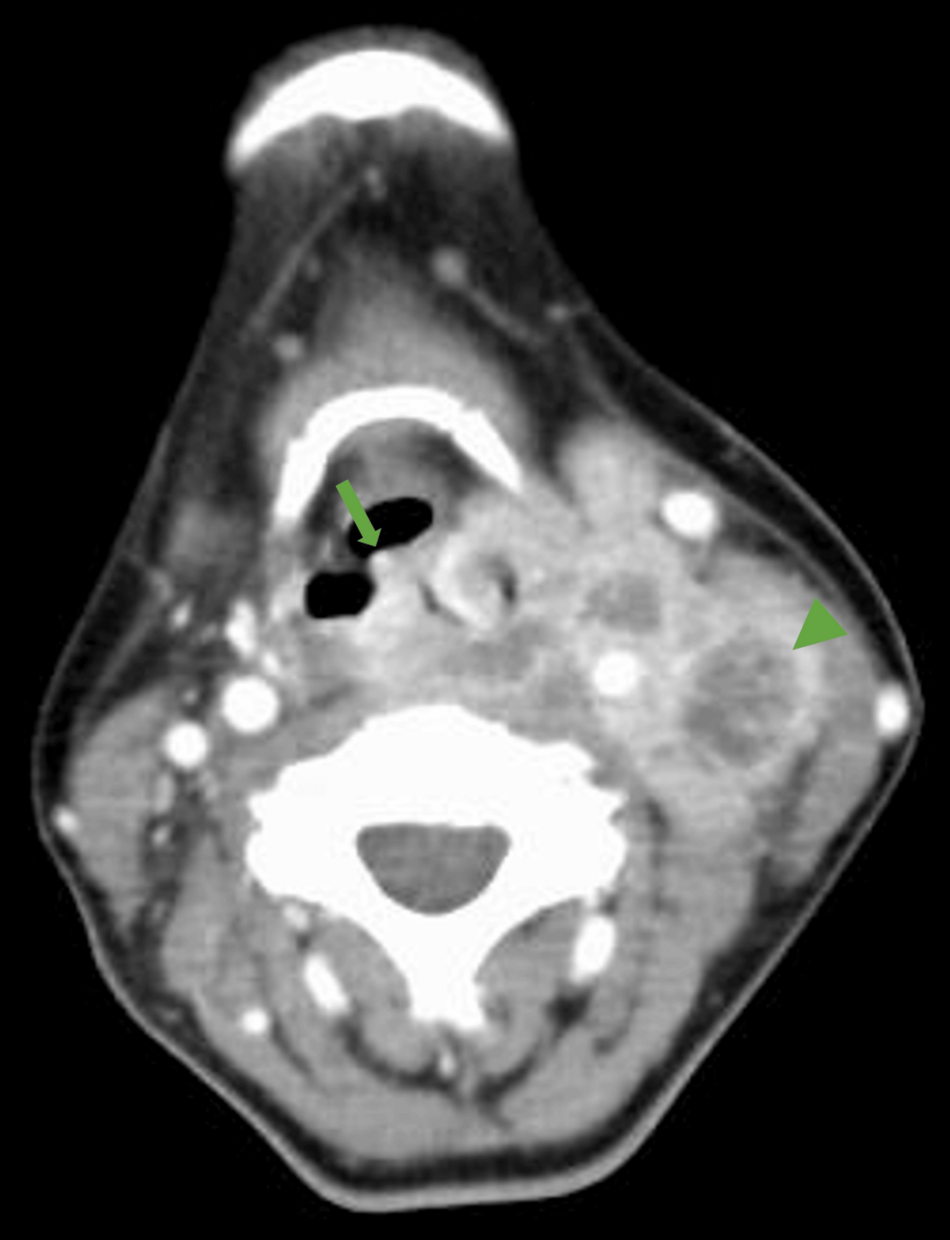
Contrast-enhanced computed tomography of the neck Contrast-enhanced computed tomography showed a thick pharyngeal wall (arrow) and lymphadenopathy with irregular nodular borders and central necrosis (arrowhead) at the level of the pre-epiglottic space.

HPV polymerase chain reaction (PCR) testing was negative. CECT from the neck to the pelvic region revealed wall thickening in the left arytenoid region and multiple lymphadenopathies with irregular nodular borders and central necrosis, indicating potential malignant changes (Figure 2). The CECT imaging also indicated liver metastases, invasion of the cervical spine, and internal jugular vein involvement.

Nasopharyngoscopy revealed a mass on the left lateral and posterior walls of the oropharynx with imaging features suggestive of airway compromise. No abnormal findings were observed in the nasal or oral cavities, and the laryngeal structures appeared uninvolved. Given the imminent risk of obstruction confirmed through both imaging and endoscopy, along with clinical signs such as progressively worsening dyspnea, especially upon neck flexion, hoarseness, and stridor, an emergency tracheostomy was promptly performed. Subsequently, gastrostomy feeding was initiated.

Histological analysis of the endoscopically biopsied oropharyngeal lesion confirmed moderately differentiated squamous cell carcinoma, leading to a diagnosis of non-HPV-associated OPSCC. Following the diagnosis, the patient underwent staging investigations, which revealed liver metastases, vertebral invasion, and internal jugular vein involvement. The clinical stage was cT4bN2M1. Due to the advanced disease stage and poor prognosis indicated by the patient’s significantly decreased performance status (Eastern Cooperative Oncology Group or ECOG 4), weight loss of 10 kg, respiratory compromise, and overall systemic frailty, she was administered palliative radiotherapy targeting the oropharyngeal mass and cervical spine for pain relief and airway symptom management. The treatment consisted of 30 Gy administered in 10 fractions over 10 days. However, the disease continued to progress rapidly. Consequently, the patient was transitioned to best supportive care for comfort-focused management.

## Discussion

OPSCC is strongly associated with chronic tobacco and alcohol use. In this case, the patient had a significant smoking history and reported occasional alcohol consumption, which are important risk factors for this malignancy [1]. She presented with symptoms that are relatively characteristic of oropharyngeal or hypopharyngeal tumors, including throat discomfort, dysphagia, hoarseness, and a rapidly growing cervical mass. Due to the insidious onset and initial mildness of such symptoms, diagnosis is often delayed, as seen in this case. Dysphagia and odynophagia are reported in approximately 41% and 24% of OPSCC cases, respectively [6], and were both present in this case. Notably, worsening dyspnea with neck flexion strongly suggested mechanical airway compromise. Contrast-enhanced CT revealed hallmark features of malignancy: irregular nodal margins, central necrosis, and asymmetric thickening of the oropharyngeal wall. These features, particularly the irregular borders and central necrosis of cervical lymph nodes, are strongly suggestive of malignant lymph node metastasis [3]. Additionally, metastatic involvement of both cervical lymph nodes and the liver, as well as direct invasion into the cervical spine and internal jugular vein, indicated both progressive local invasion and systemic involvement. HPV PCR testing yielded negative results, confirming non-HPV-associated OPSCC, which represents a rarer and more aggressive subtype of OPSCC [4]. Compared to cases involving HPV-associated tumors, non-HPV-associated cases frequently present with pain, dysphagia, and larger primary lesions, consistent with our patient’s history [2], and often present at advanced stages with smaller primary tumors and cystic lymphadenopathy [7]. Reports of acute airway compromise as the initial presentation in OPSCC are rare but notable, as seen in cases such as a giant oropharyngeal mass causing respiratory distress and challenging intubation [8,9]. Senior anesthetic and surgical teams should prepare for and intervene early in cases of airway obstruction secondary to head and neck tumors; such interventions often involve emergency tracheostomy when non-invasive methods are insufficient [10].

Herein, early airway protection via tracheostomy was essential. In this case, tracheostomy was selected over endotracheal intubation or awake fiberoptic intubation owing to the anticipated difficulty in securing the airway through conventional methods. The oropharyngeal mass had significantly distorted the upper airway anatomy, and progressive stridor suggested a high risk of sudden airway obstruction. Hence, tracheostomy under local anesthesia was considered the safest and most effective approach for protecting the airway. Given the disease severity and lack of surgical options, palliative radiotherapy was administered to alleviate local symptoms and improve quality of life, particularly in managing pain due to vertebral invasion. Although concurrent chemoradiotherapy is the standard treatment for certain stages of OPSCC, it was deemed inappropriate in this case because of the extent of metastatic disease and the patient’s compromised general condition. Palliative radiotherapy was chosen to control local symptoms, particularly pain and airway-related distress, while minimizing treatment burden. This case is particularly notable due to the acute airway compromise requiring emergency tracheostomy, which is relatively rare in OPSCC but represents a life-threatening complication. The patient presented with airway obstruction as the first clinical emergency, which is a rare and urgent manifestation of non-HPV-associated OPSCC. As mentioned before, upper airway obstruction during tumor progression is especially uncommon in patients with OPSCC and is predominantly seen in advanced stages or aggressive HPV-negative subtypes [2].

The incidence of significant airway obstruction in OPSCC is not well documented, but OPSCC itself is relatively uncommon, with an estimated global incidence of approximately 1.1 cases per 100,000 population annually [2]. The proportion of non-HPV-associated OPSCC is declining, especially in the post-HPV vaccination era. While HPV-associated OPSCC currently accounts for the majority of new cases (up to 70% in many developed regions), HPV-negative tumors are less common and tend to be more aggressive, with significantly lower five-year survival rates (<50%) compared to their HPV-positive counterparts (>80%) [2]. In this context, the current case is particularly rare and clinically significant, not only due to its HPV-negative status but also because it initially presented with airway obstruction as the first and primary clinical emergency. Such presentations are uncommon and underscore the need for prompt recognition and airway management in similar high-risk patients. Moreover, this case highlights the aggressive nature of HPV-negative oropharyngeal carcinoma and the importance of the early referral of patients with symptoms such as progressive dysphagia and cervical masses to otolaryngology specialists. In advanced-stage disease, the timely involvement of a multidisciplinary team, including oncology, otolaryngology, and palliative care, is crucial for optimal management and quality of life.

Despite a broad range of differential diagnoses, including infectious lymphadenitis (e.g., mycobacterial), granulomatous diseases such as sarcoidosis, lymphoma, and metastatic disease from an unknown primary lesion, the clinical context indicated that these diagnoses were less probable (Table 2). The absence of fever or night sweats, no history of tuberculosis exposure, and lack of generalized lymphadenopathy reduced the likelihood of infectious or granulomatous causes. Lymphoma was considered a differential diagnosis, particularly in view of the bilateral cervical lymphadenopathy and systemic symptoms. However, the asymmetric involvement, presence of necrosis on imaging, and absence of mediastinal or abdominal lymph node enlargement were not typical. Metastatic disease from an unknown primary was also considered but was less favored given the prominent oropharyngeal lesion indicated by endoscopy and imaging. Ultimately, the rapid clinical progression, marked weight loss, and hallmark CT features were most consistent with a diagnosis of primary OPSCC, which was confirmed histologically. Therefore, clinicians should maintain a high index of suspicion for OPSCC in patients with persistent pharyngeal symptoms and cervical lymphadenopathy, particularly in those with risk factors such as heavy smoking. Prompt imaging and early airway assessment can save lives in cases with signs of airway compromise.

**Table 2:** Differential diagnosis of cervical lymphadenopathy

Differential diagnosis	Reasons for exclusion
Lymphoma	Asymmetric involvement, central necrosis on imaging, no mediastinal/abdominal lymphadenopathy, or no B symptoms
Tuberculosis	No history of exposure to tuberculosis, absence of fever/night sweats, or no pulmonary findings or caseating necrosis
Sarcoidosis	No non-caseating granulomas, no chest imaging abnormalities, or lack of systemic signs
Metastatic disease from an unknown primary	Prominent oropharyngeal lesion on endoscopy and computed tomography consistent with a primary tumor

## Conclusions

This case highlights the importance of early detection and airway protection in patients with OPSCC who present with progressive pharyngeal symptoms and an enlarging neck mass. Imaging features such as nodal necrosis and asymmetric thickening should prompt urgent evaluation. In high-risk patients with cervical masses, especially those with a history of smoking, clinicians should consider malignancy during early diagnosis and promptly refer such patients to an otolaryngologist for airway assessment and management. This case also highlights the aggressive nature of HPV-negative oropharyngeal carcinoma. In advanced stages, the timely involvement of a multidisciplinary team, including oncology, otolaryngology, and palliative care, is crucial for optimal disease management and quality of life.
